# PIP_2_ corrects an endothelial Piezo1 channelopathy

**DOI:** 10.1073/pnas.2522750122

**Published:** 2025-12-23

**Authors:** Ahmed M. Hashad, Mohammad M. Abd-Alhaseeb, Xin Rui Lim, Natalia M. Mathieu, Osama F. Harraz

**Affiliations:** ^a^Department of Pharmacology, Larner College of Medicine, Vermont Center for Cardiovascular and Brain Health, University of Vermont, Burlington, VT 05405

**Keywords:** Piezo1, PIP_2_, G_q_PCR, neurovascular coupling, cerebral blood flow

## Abstract

Brain capillaries are sensors of neural activity. When a brain region is active, capillary endothelial cells (ECs) sense neuron-derived mediators and elicit a local increase in blood flow (functional hyperemia) to support the rise in metabolic needs. This hyperemic response involves a rapid electrical component and a slower chemical component that involves Gαq PCR (G_q_PCR) activation by agonists released from neurons. The intravascular forces associated with hyperemia engage mechanosensitive Piezo1-mediated signaling that serves a mechano-feedback control function to facilitate the return of elevated blood flow to basal levels. Whether G_q_PCR activity influences Piezo1 mechanosensitive signaling has not been explored, despite the potential significant implications of such crosstalk. Using patch-clamp electrophysiology and freshly isolated brain capillary ECs, we demonstrate that prostanoid or muscarinic G_q_PCR activation facilitates Piezo1 activity. Pharmacological studies revealed the involvement of Gαq and phospholipase C stimulation, as well as downstream phosphatidylinositol-4,5-bisphosphate (PIP_2_) hydrolysis in Piezo1 activation, but not signaling triggered by metabolites of PIP_2_ hydrolysis. Exogenous application of nanomolar-to-micromolar PIP_2_ suppressed Piezo1 open probability. Brain capillary ECs from mouse models of Alzheimer’s disease, cerebral small vessel disease, or Piezo1 gain-of-function mutation exhibited higher Piezo1 activity, that was corrected by exogenous ex vivo PIP_2_ application. We finally tested in vivo the hypothesis that systemic PIP_2_ administration restores functional hyperemia in EC-specific Piezo1 gain-of-function mutant mice suffering impaired blood flow. Our findings provide insights into Piezo1 channel regulation and how it affects neurovascular coupling and cerebral blood flow.

## Significance

Maintaining adequate tissue perfusion is essential for normal brain function, and its disruption is linked to many pathological conditions. Understanding the different mechanisms involved in neurovascular coupling will help identify molecular targets that can enhance brain blood flow in these conditions. The current study identifies a plasma membrane phospholipid (PIP_2_) as a possible link by which chemical signals released in the brain could modulate the activity of the brain vascular mechanosensor Piezo1. We also highlight the potential therapeutic strategy in which PIP_2_ corrects Piezo1 dysfunction and restores impaired brain blood flow.

The cardiovascular system supplies tissues and cells with their necessary nutrients and oxygen. This delivery starts with the heart pumping blood into vascular networks that include arteries, arterioles, and capillary networks which reside near all cells. The endothelium lines the lumen of all blood vessels and is crucial for blood flow regulation. Endothelial cells (ECs) sense and respond to chemical and mechanical stimuli; the failure to do so leads to pathological conditions such as hypertension and atherosclerosis (1). ECs are therefore equipped with receptors and sensors to convert stimuli into downstream—often electrical and/or Ca^2+^—signaling (2, 3). Endothelial mechanosensors include ion channels (e.g., Piezo1) and mechanosensitive receptors (e.g., G_q_PCR) (4). Piezo1 is a Ca^2+^/Na^+^-permeable channel, and its activation in ECs by force modulates Ca^2+^ signaling and the endothelial membrane potential (5–8). Mechanosensitive G_q_PCR can also be activated by force, even in the absence of prototypical receptor agonist binding (9–11). Activation of G_q_PCR stimulates phospholipase C (PLC) which hydrolyzes the membrane-bound phospholipid phosphatidylinositol 4,5-bisphosphate (PIP_2_) into inositol 1,4,5 triphosphate (IP_3_) and diacylglycerol (DAG). In the endothelium, IP_3_-mediated Ca^2+^ release and DAG-mediated activation of protein kinase C (PKC) are known modulators of several signaling pathways (12, 13).

Piezo1 channel and G_q_PCRs are functionally expressed in the brain vasculature (2, 6, 14, 15). Mechanical stimulation of CNS ECs increases endothelial Ca^2+^ transients, via Piezo1 channel activation (6). G_q_PCR activation in ECs is also critical for brain endothelial Ca^2+^ signaling. Neuron-derived receptor agonists [e.g., prostaglandin E_2_ (PGE_2_)] evoke capillary Ca^2+^ transients through IP_3_-mediated Ca^2+^ release and by facilitating Ca^2+^ influx via the vanilloid 4 transient receptor potential TRPV4 channel (14). Capillary endothelial Ca^2+^ signals activate endothelial nitric oxide (NO) synthase (eNOS) to generate the vasodilator NO (8), which relaxes some but not all capillaries (14). Piezo1 also depolarizes brain ECs and helps the return of blood flow to baseline levels after hyperemia (5). Interestingly, G_q_PCR activity in brain capillary ECs modulates ion channel activity. We have previously shown G_q_PCR influences brain blood flow by altering the activity of the inwardly rectifying K^+^ channel Kir2.1 and TRPV4 (15, 16). Considering the mechanosensitive properties of some G_q_PCRs along with G_q_PCR’s ability to regulate endothelial ion channels, we set out to explore whether—and if so how—G_q_PCR signaling modulates Piezo1 activity. This is a gap with high stakes, given the centrality of brain vascular mechanosensation to blood flow regulation and supporting neuronal function.

Here, we used single-channel electrophysiology and in vivo brain blood flow imaging to tackle this question. We demonstrate that G_q_PCR activation enhances Piezo1 function in brain capillary ECs and that this enhancement was predominantly mediated by the depletion of PIP_2_. We further observed an upregulation of capillary EC Piezo1 function in different disease mouse models, and importantly, PIP_2_ corrected this Piezo1 channelopathy and the associated cerebral blood flow (CBF) deficits. Collectively, the current study provides insights into how G_q_PCR activation influences the endothelial mechanosensor Piezo1.

## Results

### G_q_PCR Activation Enhances Piezo1 Current in Capillary ECs.

**Fig. 1.**
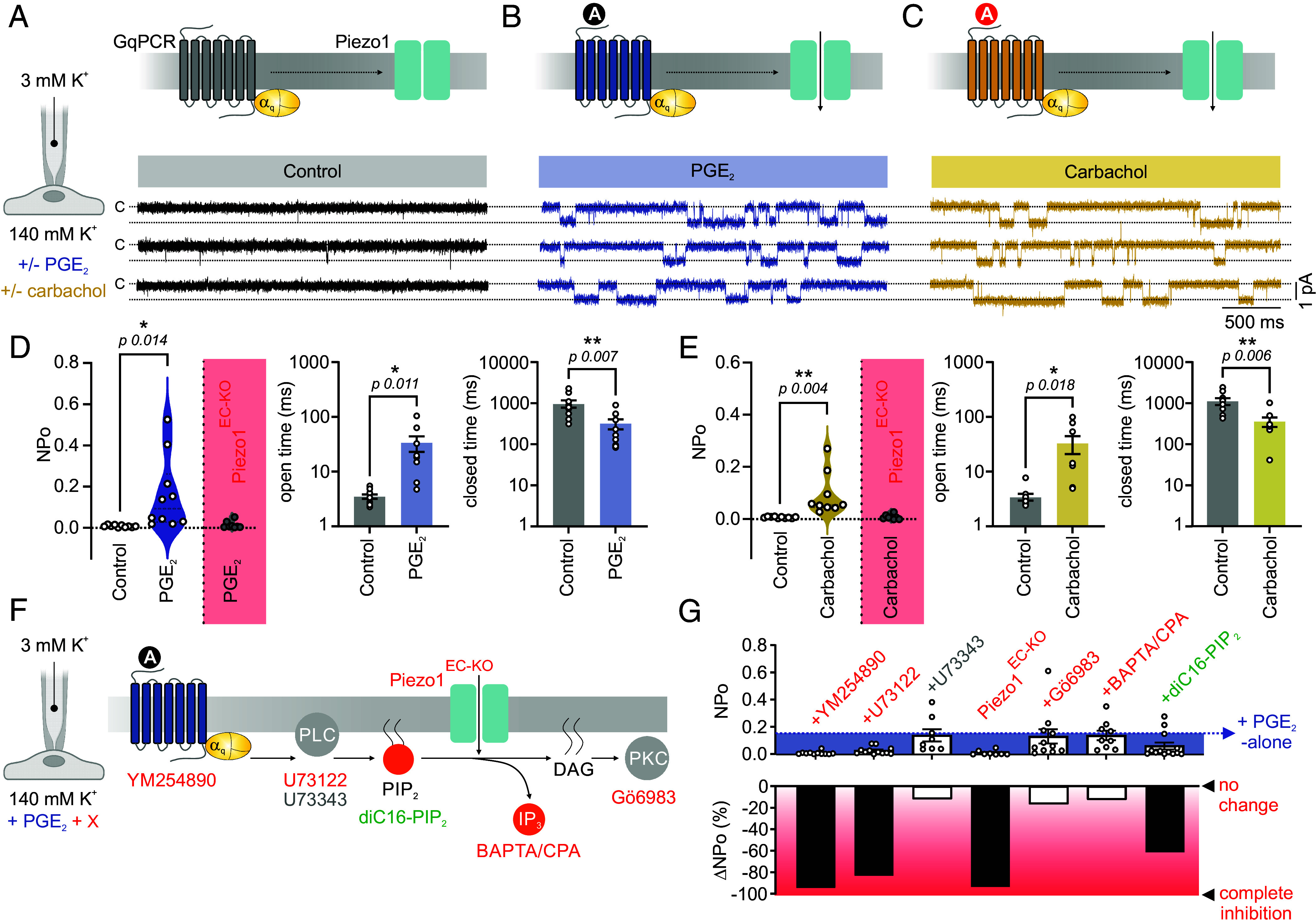
G_q_PCR activation enhances capillary endothelial Piezo1 channel activity. (*A*) Schematic diagrams and representative traces of Piezo1 current recorded from a freshly isolated capillary EC from the somatosensory cortex of a C57BL/6J mouse. Recordings were made in the cell-attached configuration, and EC patches were held at −50 mV. (*B* and *C*) Similar to *A*, recordings were made in the presence of PGE_2_ (2 µM, *B*) or carbachol (10 µM, *C*), added to the bath solution for ≥20 min before recording. (*D*) Averaged data of Piezo1 NP_O_, open time, and closed time in the absence or presence of PGE_2_ (n = 10/6 ECs/C57 mice control, n = 10/7 PGE_2_). ECs from Piezo1^EC-KO^ mice were subjected to the same conditions (n = 8/4). (*E*) Scatter plots of Piezo1 activity with and without carbachol (n = 10/5 control, n = 9/4 carbachol, n = 8/4 Piezo1^EC-KO^+carbachol). (*F*) Schematic demonstrating the experimental conditions and the signaling pathway downstream of G_q_PCR activation. Pharmacological interventions used to block different components of the pathway are highlighted. (*G*) *Top*: Summary data showing Piezo1 NP_O_ in response to PGE_2_ in the presence of YM254890 (1 µM, n = 11/4), U73122 (10 µM, n = 12/3), U73343 (10 µM, n = 8/3), Gö6983 (1 µM, n = 11/4), CPA (30 µM)/BAPTA (10 µM) (n = 9/4), diC16-PIP_2_ (10 µM, n = 13/4), or in Piezo1^EC-KO^ ECs (n = 8/4). The blue arrow shows PGE_2_ effect under control conditions. Drugs were added to the bath for ≥20 min before recording. *Bottom*: Corresponding percentage inhibition of the PGE_2_ effect. All data are expressed as mean ± SEM. Unpaired Student’s *t* test was used in *D* and *E* (*P < 0.05, **P < 0.01).

Several G_q_PCRs in brain capillary ECs are activated by receptor agonists released from nearby active neurons (14, 17). Previous reports, including ours, have shown that G_q_PCR signaling modulates endothelial ion channels, including those in brain capillaries (15, 16). The mechanosensor Piezo1 is functionally expressed in brain capillary ECs (6), but it is unclear whether G_q_PCR activity alters Piezo1 function. To explore this, we used patch-clamp electrophysiology to measure Piezo1 activity in freshly isolated C57BL/6J brain capillary ECs in the cell-attached configuration. The open probability (NP_O_) of Piezo1 in EC patches held at −50 mV was very low (0.008 ± 0.002) (Fig. 1*A*). Incubating ECs with the receptor agonist PGE_2_—a neuronally released agent previously shown to activate the G_q_ protein–coupled prostanoid EP1 receptor in ECs (14, 18)—caused a dramatic (~20-fold) increase in Piezo1 NP_O_. To test whether this observation was limited to prostanoid receptor activation, we used the muscarinic G_q_PCR agonist carbachol and observed significant enhancement of Piezo1 activity (Fig. 1 *A*–*C*). In addition to enhanced NP_O_, both PGE_2_ and carbachol increased the open time and decreased the closed time of Piezo1 current (Fig. 1 *D* and *E*). To confirm that the observed currents were mediated by Piezo1, we tested PGE_2_ and carbachol on ECs isolated from endothelial-specific Piezo1 knockout mice (Piezo1^EC-KO^). Both agonists failed to evoke inward currents when Piezo1 was genetically ablated (Fig. 1 *D* and *E*). These results suggest that endothelial G_q_PCR activation promotes Piezo1 activity.

### G_q_PCR Signaling Enhances EC Piezo1 Via PIP_2_ Depletion.

G_q_PCR signaling activates PLC which hydrolyzes PIP_2_ into IP_3_, which enhances Ca^2+^ release, and DAG, which activates PKC. To dissect the signaling components involved in G_q_PCR-mediated Piezo1 activation, we employed pharmacological tools along with electrophysiology to specifically target different signaling steps (Fig. 1*F*). First, we confirmed that PGE_2_ signals through Gα_q_ protein, since PGE_2_ failed to enhance Piezo1 activity in ECs pretreated with the Gα_q_ inhibitor YM254890. Inhibition of PLC with U73122, but not its inactive analog U73343, similarly prevented PGE_2_-mediated Piezo1 current indicating that channel activation requires G_q_/PLC signaling (Fig. 1 *F* and *G*). PLC hydrolyzes PIP_2_ into the active metabolites IP_3_ and DAG. Since IP_3_ transduces by modulating intracellular [Ca^2+^], we simultaneously inhibited the sarcoplasmic/endoplasmic reticulum Ca^2+^ ATPase (SERCA) pump using cyclopiazonic acid—to deplete intracellular Ca^2+^ stores—along with rapid cytoplasmic Ca^2+^ chelation using BAPTA—to abort Ca^2+^ release induced signaling—but still failed to abolish the PGE_2_-evoked increase in Piezo1 activity. Additionally, PGE_2_ enhanced Piezo1 activity even in the presence of the PKC inhibitor Gö6983 (Fig. 1 *F* and *G*). Our data suggest that the effect of PGE_2_ on Piezo1 involves G_q_/PLC signaling but does not primarily depend on IP_3_ or DAG formation. Since the latter are breakdown products of PIP_2_ hydrolysis and since PIP_2_ is a key regulator of ion channels (19), we next sought to test whether PIP_2_ influences Piezo1 function. To determine whether PIP_2_ depletion increases Piezo1 activity, we tested the effects of PGE_2_ in ECs incubated with PIP_2_. In the presence of the water-soluble diC16-PIP_2_ sodium salt, Piezo1 activation by PGE_2_ was significantly attenuated (Fig. 1 *F* and *G*), suggesting an inhibitory effect for PIP_2_ on Piezo1 that is relieved by G_q_PCR activation.

### PIP_2_ Suppresses Piezo1 Function in Brain Capillary ECs.

**Fig. 2.**
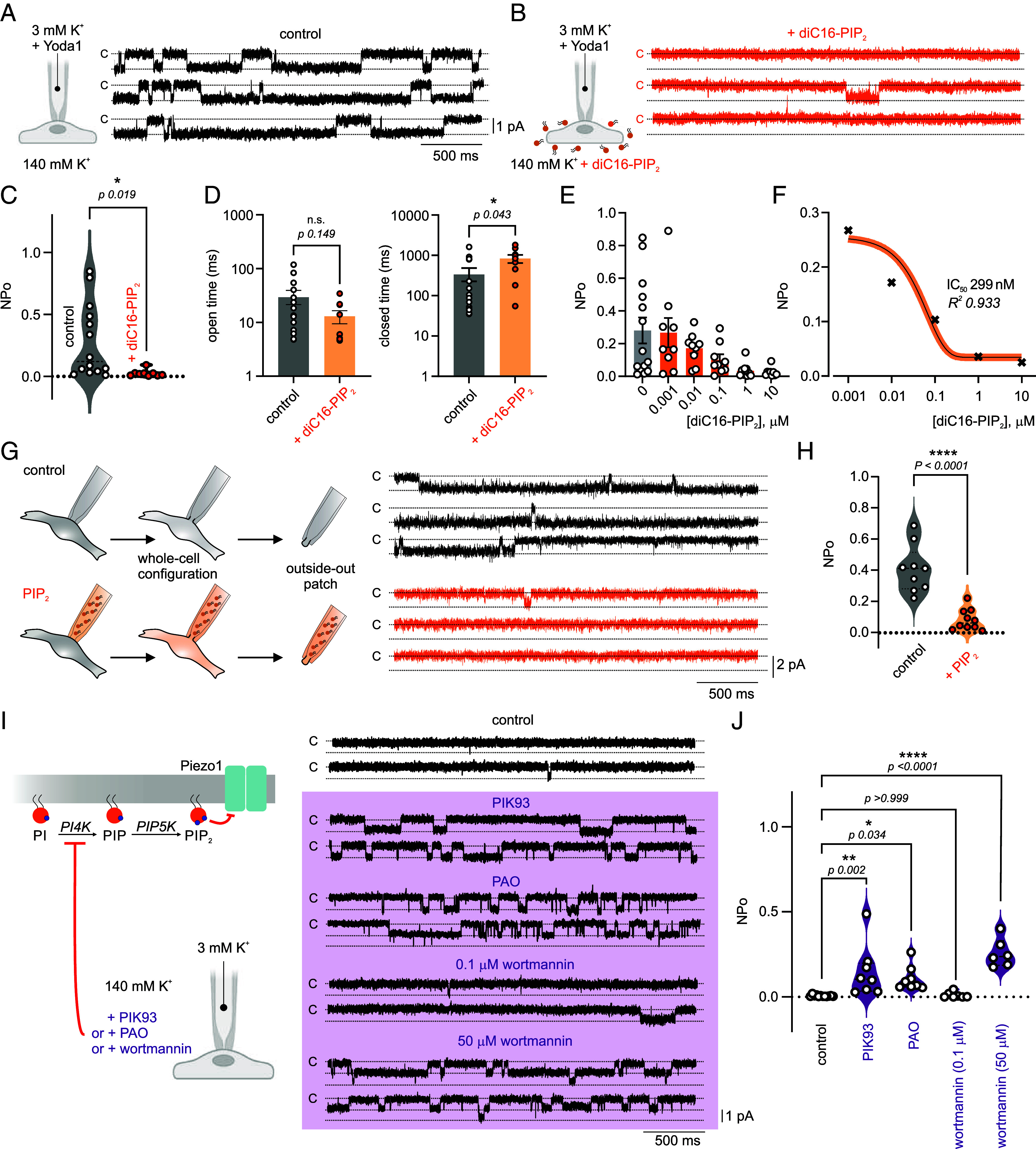
PIP_2_ inhibits Piezo1 current in brain capillary ECs. Representative traces of Piezo1 currents recorded from capillary ECs from in the absence (*A*) or the presence of diC16-PIP_2_ (10 µM) in the bath (*B*). Yoda 1 (5 µM) was added to pipette solution, and recordings were made at −50 mV. (*C* and *D*) Averaged scatter plots of Piezo1 NP_o_, open and closed times in control condition (n = 14/10) and with diC16-PIP_2_ (n = 9/7). Unpaired Student’s *t* test (*P < 0.05, n.s., denotes not significant). (*E* and *F*) Concentration response curve and sigmoidal fitting of Piezo1 activity in the presence of varying concentrations of diC16-PIP_2_ (1 nM, n = 9/5; 10 nM n = 9/4; 100 nM n = 9/4; 1 µM n = 10/3; 10 µM n = 9/6). IC_50_ denotes half maximal inhibitory concentration. (*G*) Outside-out patch-clamp experiments with pipette solutions supplemented or not supplemented with 10 µM diC16-PIP_2_. Currents were recorded at −70 mV in the absence of Yoda1. (*H*) Averaged scatter plots of Piezo1 NP_o_ in control condition (n = 9/4) and with PIP_2_ (n = 10/4). Unpaired Student’s *t* test (****P < 0.0001). (*I*) Schematic demonstrating PIP_2_ synthesis pathway and representative traces of control Piezo1 currents and currents in the presence of the PI4K inhibitors PIK93 (100 nM), phenylarsine oxide (PAO, 10 µM), or wortmannin (50 µM). Lower concentration wortmannin (0.1 µM) was also tested. ECs were incubated with kinase inhibitors for ≥20 min before recording, and Yoda1 was not used. (*J*) Summary data showing Piezo1 NP_O_ in absence (n = 12/4) and presence of PIK93 (n = 8/6), PAO (n = 8/5), wortmannin 0.1 µM (n = 7/5), or 50 µM (n = 6/5). One-way ANOVA followed by Dunnett’s multiple comparisons test. Data are expressed as mean ± SEM (*P < 0.05, **P < 0.01, ****P < 0.0001).

The G_q_PCR/PLC pathway governs PIP_2_ levels in the inner leaflet plasma membrane. G_q_PCR activation rapidly (within seconds) depletes ~90% of cellular PIP_2_ in overexpression systems (19–22), though such reduction in native cells could be less profound and is highly receptor-specific (23, 24). Our findings (Fig. 1) indicated that G_q_PCR agonists increase Piezo1 activity, presumably by depleting PIP_2_. We sought next to characterize Piezo1 modulation by PIP_2_ without G_q_PCR activation. In the absence of G_q_PCR agonists, Piezo1 NP_O_ is extremely low (Fig. 1 *A*–*E*). Therefore, the selective Piezo1 activator Yoda1 (5 µM) was used to augment and measure Piezo1 activity in the absence or the presence of PIP_2_. As expected, Yoda1 increased Piezo1 activity (NP_O_ 0.28 ± 0.08), and ECs incubation with 10 µM diC16-PIP_2_ profoundly inhibited Piezo1 function (NP_O_ 0.03 ± 0.01) and increased Piezo1 closed time (Fig. 2 *A*–*D*). Under physiological conditions, PIP_2_ is predominantly located in the plasma membrane, representing ~1% of the acidic lipids in the cell. It is therefore estimated that if total cellular PIP_2_ was dissolved in the cytoplasm it would be equivalent to ~5 to 10 µM solution (19, 25, 26). We used 10 µM PIP_2_ and observed Piezo1 inhibition in brain capillaries (Fig. 2*C*), but to determine whether lower concentrations inhibit channel function, we measured Piezo1 currents in the presence of varying PIP_2_ concentrations (1 nM to 10 µM) (Fig. 2 *E* and *F*). Piezo1 NP_O_ in the presence of 1 nM diC16-PIP_2_ was similar to control values in the absence of PIP_2_ (0.27 ± 0.09 and 0.28 ± 0.08, respectively), and concentrations of 10 nM or above inhibited Piezo1 current. We estimated using a concentration–response curve that the [PIP_2_] needed to inhibit 50% of the maximal current (i.e., IC_50_) was ~300 nM. These data suggest that capillary EC Piezo1 channel is tonically inhibited by PIP_2_ (Fig. 2 *E* and *F*), similar to our previous observation of tonic inhibition of TRPV4 channel (16).

Physiologically, PIP_2_ is most abundant in the inner leaflet of the plasma membrane. Externally applied PIP_2_ inhibited Piezo1 currents (Fig. 2*C*), but whether this action was due to PIP_2_ in the outer leaflet or due to flipping into the inner leaflet is unclear. We have previously demonstrated that externally applied PIP_2_ enhances inward-rectifier Kir2.1 channels (27, 28), but there is evidence that intracellular and extracellular PIP_2_ can have different effects on the same ion channel (29, 30). To test whether PIP_2_ applied intracellularly inhibits Piezo1 in ECs, we next performed outside-out patch-clamp experiments with PIP_2_ included in the pipette. Piezo1 NP_O_ was significantly lower in outside-out patches when PIP_2_ was added to the pipette solution (0.08 ± 0.02) compared to control patches (0.41 ± 0.05) (Fig. 2 *G* and *H*). This observation is consistent with recent evidence demonstrating that PIP_2_ inhibits Piezo1 current when applied intracellularly in outside-out HEK293 patches (31). In summary, our observations show that PIP_2_ applied extracellularly or intracellularly inhibits Piezo1 activity in ECs.

### Inhibition of PIP_2_ Synthesis Enhances Piezo1 Activity.

The level of PIP_2_ in the plasma membrane is dynamically regulated and determined by the balance between ongoing synthesis and hydrolysis. PIP_2_ is synthesized in a 2-step phosphorylation process of the precursor phosphatidylinositol (PI) (19). Phosphatidylinositol 4-kinase (PI4K) converts PI into phosphatidylinositol 4-phosphate (PI(4)P), which is subsequently phosphorylated by phosphatidylinositol 4-phosphate 5-kinase (PIP5K) to form PI(4,5)P_2_ (i.e., PIP_2_, Fig. 2*I*). PI4K function is the rate limiting step in PIP_2_ synthesis (32–34), that is why inhibiting PI4K shifts the dynamic PIP_2_ synthesis/hydrolysis balance and reduces [PIP_2_] in the plasma membrane (15). We hypothesized that PI4K inhibition will enhance Piezo1 function. The incubation of capillary ECs with the PI4K inhibitor PIK93 increased Piezo1 NP_o_, even in the absence of Yoda1. Phenylarsine oxide (PAO) is a structurally unrelated PI4K inhibitor that similarly enhanced Piezo1 currents (Fig. 2 *I* and *J*). To corroborate the evidence presented with PIK93 and PAO, we next used the inhibitor wortmannin at two different concentrations (0.1 and 50 µM), as we did before (16). At a concentration below that necessary to inhibit PI4K, but sufficient to suppress phosphoinositide 3-Kinase (PI3K), wortmannin (0.1 µM) failed to enhance currents in ECs. However, 50 µM wortmannin significantly enhanced NP_O_ of an inward current with a slope conductance consistent with Piezo1 (~21 pS) (Fig. 2 *I* and *J* and *SI Appendix*, Fig. S1). These data collectively demonstrate that Piezo1 is inhibited by intracellular PIP_2_ and that the dynamic regulation of PIP_2_ levels influences Piezo1 function in brain capillaries.

### PIP_2_ Does Not Affect EC Piezo1 Mechanosensitivity.

PIP_2_ could directly interact with Piezo1 residues and regulate channel function (31, 35–37). By incorporating into the inner leaflet of the plasma membrane, PIP_2_ could also modify membrane fluidity (38, 39) and therefore Piezo1 activity. We sought to investigate the impact of PIP_2_ on Piezo1 mechanosensitivity in brain capillary ECs by subjecting endothelial patches to negative pressure steps (−5, −10, −15, −20, −25 mmHg) in the presence or absence of diC16-PIP_2_. Negative pressures elicited Piezo1 openings in both conditions, with an overall lower NP_O_ in the presence of diC16-PIP_2_ (*SI Appendix*, Fig. S2 *A*–*D*). Boltzmann sigmoidal function showed that the pressure evoking half-maximal activation (P_50_) was similar whether PIP_2_ was present (−4.9 mmHg) or absent (−4.8 mmHg) (*SI Appendix*, Fig. S2 *E*–*H*). While additional mechanosensitivity testing is warranted, our data robustly show that PIP_2_ suppresses endothelial Piezo1 channel openings.

### PIP_2_ Rescues Piezo1 Current Dysregulation in Disease.

**Fig. 3.**
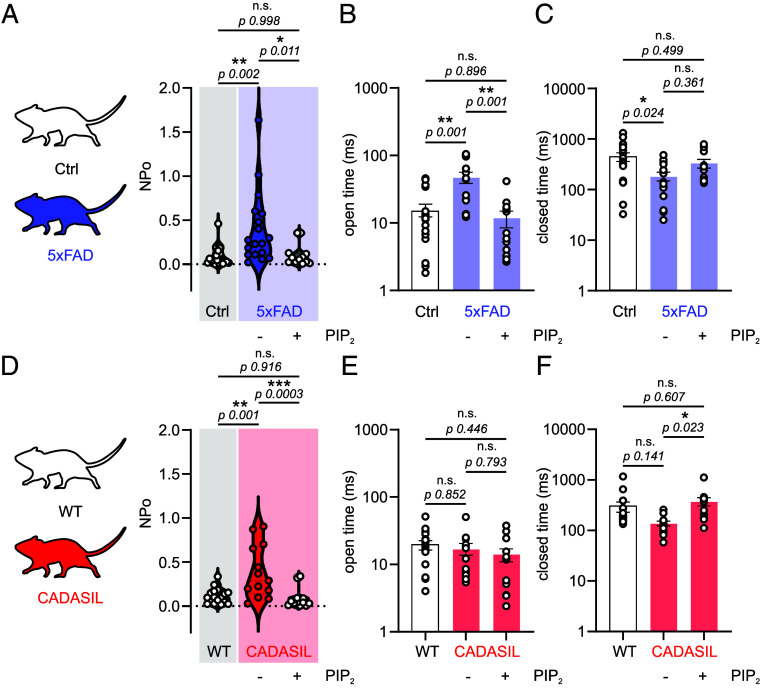
PIP_2_ corrects brain endothelial Piezo1 channelopathy. (*A*) Piezo1 activity in brain capillary ECs from control mice (n = 21/7) or 5xFAD mice, where 5xFAD ECs were treated (n = 12/4) or untreated (n = 18/7) with diC16-PIP_2_ (10 µM). Patches were held at −50 mV, and Yoda1 (5 µM) was in the pipette solution. (*B* and *C*) Open and closed times in the three groups described in *A*. (*D*–*F*) Averaged data of Piezo1 NP_O_ (*D*), open time (*E*), and closed time (*F*) in control wild-type (WT) mice (n = 17/4) and ECs from CADASIL mice with (n = 14/6) or without (n = 13/7) diC16-PIP_2_ (10 µM). One-way ANOVA followed by Tukey’s multiple comparisons tests were used in all panels (*P < 0.05, **P < 0.01, ***P < 0.001, n.s., denotes not significant).

PIP_2_ levels are disrupted in multiple diseases. For instance, phosphoinositide abundance and PLC signaling are dysregulated in the brains of Alzheimer’s disease (AD) patients (40–43). Amyloid β peptides, that accumulate in AD brains, interfere with PIP_2_ synthesis and lower its levels in the brain (44, 45). Since PIP_2_ suppressed Piezo1 function (Fig. 2), we hypothesized that capillary Piezo1 function would be enhanced in diseases where PIP_2_ levels are compromised. We previously reported in a mouse model of familial AD (5xFAD) that Kir2.1 activity is reduced along with brain blood flow deficits, both of which were reversed with PIP_2_ (28). Here, we isolated capillary ECs from 5xFAD mice or their control to measure Piezo1 activity. Electrophysiological measurements revealed significantly higher NP_O_, higher open time, and lower closed time in 5xFAD ECs compared with cells from littermate controls (Fig. 3 *A*–*C*). Incubation of 5xFAD ECs with diC16-PIP_2_ significantly suppressed channel NP_O_ and open time to levels comparable with controls (Fig. 3 *A* and *B*). These data reveal a Piezo1 channelopathy (i.e., upregulation) in an AD mouse model and further show the ability of PIP_2_ to correct this dysfunction.

Cerebral small vessel disease (cSVD) represents another pathology where capillary Kir2.1 is dysfunctional in a PIP_2_-dependent manner (27, 46). We therefore hypothesized that Piezo1 function is altered in a cSVD mouse model. The *TgNotch3^R169C^* mouse model of cerebral autosomal dominant arteriopathy with subcortical infarcts and leukoencephalopathy (CADASIL) was used, and the wild-type *TgNotch3^WT^* (WT) mice were used as controls. Piezo1 NP_O_ in CADASIL capillary ECs was significantly higher (0.39 ± 0.08) than those in WT ECs (0.11 ± 0.02) (Fig. 3*D*). Incubation of CADASIL capillaries with diC16-PIP_2_ (10 µM) significantly suppressed Piezo1 NP_O_ (0.09±0.03) and increased channel closed time to *TgNotch3^WT^* levels (Fig. 3 *D*–*F*). Considering that Piezo1 NP_O_ was low in 5xFAD control ECs (Fig. 3*A*) and *TgNotch3^WT^* ECs (Fig. 3*D*), we only tested the effects of PIP_2_ on ECs from 5xFAD and CADASIL mice. Taken together, these findings reemphasize an inhibitory action of PIP_2_ on Piezo1 activity and importantly point out the potential for PIP_2_ to correct Piezo1 channelopathies.

### PIP_2_ Corrects Piezo1 Current in Piezo1^EC-Mutant^ ECs.

**Fig. 4.**
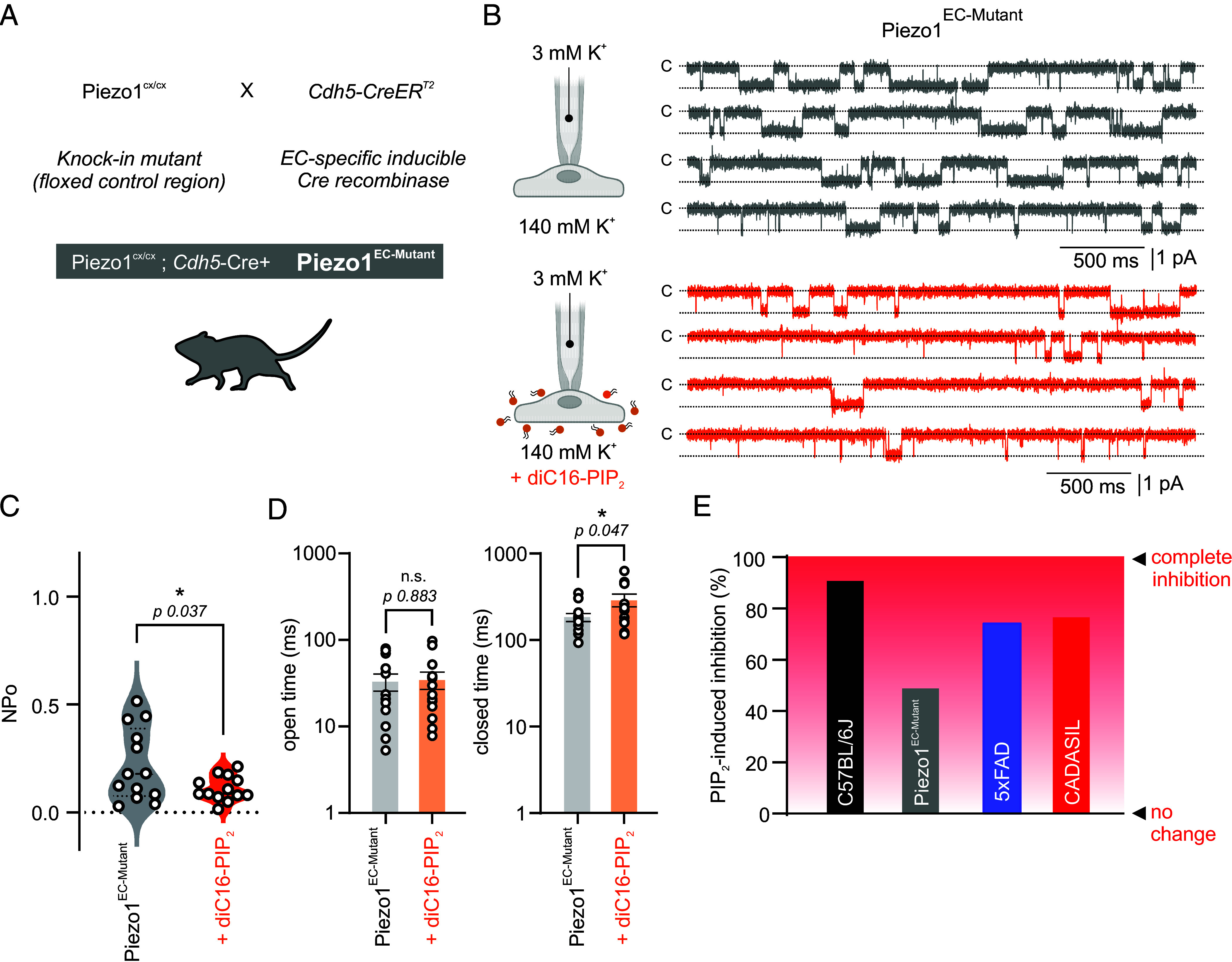
PIP_2_ normalizes endothelial Piezo1 activity in Piezo1^EC-Mutant^ gain-of-function mice. (*A*) Schematic of the cross-breeding used to generate inducible, EC-specific Piezo1 gain-of-function mice (Piezo1^EC-Mutant^). (*B*) Representative traces of Piezo1 currents in capillary ECs in the absence and the presence of diC16-PIP_2_ (10 µM). (*C* and *D*) Averaged data of Piezo1 NP_O_, open time, and closed time recorded from Piezo1^EC-Mutant^ capillary ECs without (n = 13/4) or with diC16-PIP_2_ (n = 14/4). ECs were held at −50 mV, and recordings were made in the absence of Yoda1. Unpaired Student’s *t* test was used in *C* and *D* (*P < 0.05). (*E*) The extent of PIP_2_-mediated inhibition of Piezo1 current (relative to no PIP_2_ respective values) in ECs from C57BL/6J, Piezo1^EC-Mutant^, 5xFAD, or CADASIL mice.

Several human *PIEZO1* mutations have been reported (47–49). An aggressive gain-of-function *PIEZO1* mutation (R2456H), causing hemolytic anemia in hereditary xerocytosis patients, is linked to loss of channel inactivation, sensitization to mechanical force, and ultimately more cation influx (47, 50, 51). We have recently engineered and characterized an endothelial-specific Piezo1 mutant mouse model [hereafter, Piezo1^EC-Mutant^ (5)] that carries the mouse *Piezo1* point mutation R2482H, equivalent to R2456H in human xerocytosis patients (Fig. 4*A*). Brain capillary ECs from Piezo1^EC-Mutant^ mice exhibit higher Piezo1 activity compared to ECs from Cre-negative control littermates (5). Since PIP_2_ inhibited Piezo1 currents in 5xFAD and CADASIL ECs (Fig. 3), we sought to investigate the effect of PIP_2_ on Piezo1^EC-Mutant^ ECs. These experiments were notably performed in the absence of Yoda1 to avoid maxing out Piezo1 openings. Piezo1 NP_O_ was 0.22 ± 0.05 in the absence of PIP_2_ in Piezo1^EC-Mutant^ ECs, and cells incubated with diC16-PIP_2_ had significantly lower activity (0.11 ± 0.02) (Fig. 4 *B* and *C*). Closed times increased in the presence of PIP_2_, but open times were unaffected (Fig. 4*D*). Interestingly, the inhibition by PIP_2_ was much stronger in ECs from C57BL/6J (91%), 5xFAD (77%), and CADASIL (74%) mice, compared with ECs from Piezo1^EC-Mutant^ mice (50%) (Fig. 4*E*). Collectively, our observations indicate that PIP_2_ inhibits mutant Piezo1 channel and could further suggest that the Piezo1 mutation interferes with PIP_2_/Piezo1 interaction, consistent with recent structural evidence of a critical role for the human R2456 residue in anchoring lipids at the channel pore (51).

### PIP_2_ Rescues Functional Hyperemia in Piezo1^EC-Mutant^ Mice.

**Fig. 5.**
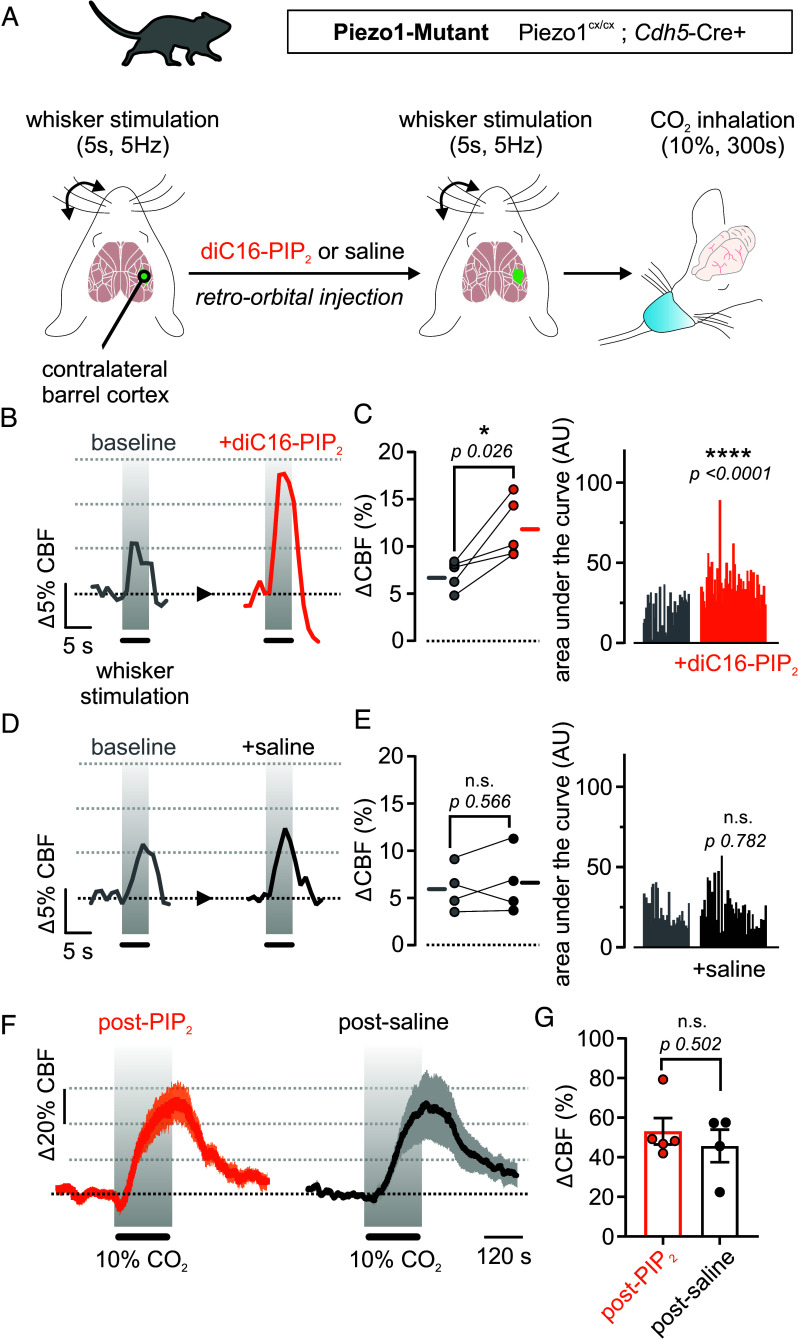
PIP_2_ enhances FH in Piezo1^EC-Mutant^ mice. (*A*) An illustration depicting the experimental protocol used to measure FH in Piezo1^EC-Mutant^ mice. We measured CBF changes in response to whisker stimulation or CO_2_ inhalation using LSCI and thinned skulls mice. (*B*) Representative traces in response to air puffs (5 s, 5 Hz) in Piezo1^EC-Mutant^ mice before and after retro-orbital injection with diC16-PIP_2_ (0.5 mg/Kg). (*C*) Maximal responses and area under the curve histograms of FH responses in Piezo1^EC-Mutant^ mice before and after PIP_2_ injection (n = 5 mice, Paired Student’s *t* test). (*D* and *E*) Similar to *B* and *C* but with saline injection (n = 4 mice, Paired Student’s *t* test). (*F* and *G*) Averaged traces and CBF responses to hypercapnia in Piezo1^EC-Mutant^ mice injected with diC16-PIP_2_ (n = 5) or saline (n = 4) (unpaired *t* test). Data are expressed as mean ± SEM (*P < 0.05).

Enhanced Piezo1 activity in Piezo1^EC-Mutant^ mice impaired the hyperemic response to somatosensory stimulation (functional hyperemia) (5). Building on previous findings and the results presented here, we hypothesized that PIP_2_ could ameliorate functional hyperemia deficits in Piezo1^EC-Mutant^ mice. Laser speckle contrast imaging (LSCI) was used to monitor CBF and measure hyperemic responses in the barrel cortex in response to whisker stimulation (air puffs; 5 s, 5 Hz) in anesthetized mice (Fig. 5*A*). Functional hyperemia responses were recorded at baseline and after systemic injection of diC16-PIP_2_ (0.5 mg/kg weight, retro-orbital) or vehicle (saline). PIP_2_ injection increased the maximal functional hyperemia response from 7.1 ± 0.7% to 11.8 ± 1.4% (Fig. 5 *B* and *C*), but saline injection had no effect (from 6.0 ± 1.2% to 6.6 ± 1.7%) (Fig. 5 *D* and *E*). Similar improvements were observed when whiskers were stimulated for 10 s (*SI Appendix*, Fig. S3). Notably, we previously demonstrated that PIP_2_ had no effect in mice with normal hyperemia (27, 28). Following the whisker stimulation after PIP_2_ or saline injection, mice were subjected to a hypercapnic challenge (10% CO_2_ inhalation for 5 min). Hyperemic responses to inhaled CO_2_ were comparable between both PIP_2_- and saline-treated Piezo1^EC-Mutant^ mice (Fig. 5 *F* and *G*). In summary, these observations demonstrate the effectiveness of PIP_2_ to correct a CBF deficit in a mouse model of Piezo1 channelopathy.

## Discussion

Brain capillary networks play a pivotal role in matching neuronal demands with adequate blood supply (52, 53). This role depends on the ability of ECs to sense and respond to different stimuli. Neuron-derived factors, such as K^+^ and G_q_PCR agonists, modulate endothelial ion channel activity directly or indirectly, thus initiating blood flow changes (14, 15, 54, 55). The consequent change in blood flow engages endothelial mechanosensors that fine-tune the delivery process. Brain capillary mechanosensors include Piezo1 and further extend to mechanically activated G_q_PCRs (4). Despite the central role of endothelial G_q_PCRs in CBF regulation (14, 56), it remains elusive whether G_q_PCR signaling modulates Piezo1 function in the brain. Here, we demonstrate that G_q_PCR stimulation augments (or disinhibits) Piezo1 activity by depleting the membrane phospholipid PIP_2_. We also highlight the potential of PIP_2_ as a means to normalize Piezo1 dysfunction and correct CBF deficit during disease. Collectively, these insights will advance our understanding of CBF regulation and dysregulation.

Endothelial G_q_PCR activation plays a crucial role in CBF regulation (14, 15, 56). Neuronal activity engages G_q_PCR signaling and the ensuing Ca^2+^ signals—via IP_3_-mediated Ca^2+^ release and amplified through TRPV4-mediated Ca^2+^ influx—facilitate the synthesis of the potent vasodilator nitric oxide (NO) and local blood flow changes in affected capillary branches (14). Brain EC Gα_q/11_ is also critical for hypercapnia-induced NO release and hyperemia (56). While neuron-derived receptor agonists are implicated in driving endothelial G_q_PCR-mediated signaling (14), several G_q_PCRs are metabotropic mechanosensors that activate independently of ligand binding. GPR68 is an endothelial G_q_PCR that mediates endothelial shear stress responses via NO production in the peripheral vasculature (9). While little is known about GPR68 in the brain endothelium, it has been recently suggested that it does not contribute to CO_2_-induced cerebrovascular responses (56). Nonetheless, mechano-activation of G_q_PCR presents a mechanism by which endothelial G_q_PCR signaling could engage in response to functional hyperemia. We propose different scenarios, presumably with distinct temporal characteristics, by which intravascular forces affect capillary Piezo1 signaling: Force directly activates Piezo1, and G_q_PCR signaling indirectly disinhibits Piezo1. G_q_PCR activation could be a means for Piezo1 signal amplification, by elevating the gain of Piezo1 opening and recruiting otherwise tonically inhibited channels (Fig. 1). We speculate that strategic positioning of G_q_PCRs and the consequent potential for ambient PIP_2_ depletion could create hot spots for Piezo1 activity augmentation during functional hyperemia. This speculation awaits further testing to examine G_q_PCR/Piezo1 colocalization.

Activation of G_q_PCR by ligands or shear stress activates PLC with subsequent hydrolysis of PIP_2_ into IP_3_ and DAG, metabolites that modulate several cellular functions via the Ca^2+^ release and PKC activation, respectively. In freshly isolated capillary ECs, inhibition of either pathway failed to prevent G_q_PCR-induced activation of Piezo1 (Fig. 1). It is noteworthy that recent findings using cultured ECs demonstrated that protein kinase A (PKA) and PKC phosphorylate Piezo1 and slow channel inactivation causing an overall increase in Piezo1 activity (57). These findings do not explain our observations that G_q_PCR agonists enhanced Piezo1 activity in brain ECs, highlighting possible differences in channel modulation by PKC in these two conditions. Phosphorylation of Piezo1 by PKC and PKA, however, could be mechanisms by which GPCRs signaling through Gαq or Gαs, respectively, can modulate endothelial Piezo1 function. In support of our findings, recent molecular dynamics simulations and electrophysiological recordings revealed that PIP_2_ binding suppresses Piezo1 activity and that mutations interfering with this binding enhance Piezo1’s chemical and mechanical activation (31). This study further showed that PIP_2_ depletion increased the channel’s sensitivity to membrane tension in outside-out patches of heterologous expression systems (31). The latter experimental conditions are distinct from ours, which could explain why we observed no difference in Piezo1 sensitivity to mechanical activation in the presence of PIP_2_. Nonetheless, additional testing—in native cells, using physiological mechanical stimuli—is warranted to determine whether PIP_2_ affects brain endothelial Piezo1 mechanosensitivity.

Despite comprising <1% of total membrane phospholipids, PIP_2_ modulates a plethora of ion channels, including those in capillary ECs (19). We previously showed that PIP_2_ is essential for maintaining Kir2.1 activity and retrograde electrical signaling (15). Additionally, PIP_2_ depletion downstream of G_q_PCR activation disinhibits TRPV4 channel and increases the channel open probability by several folds (16). Our data here indicate that Piezo1 channels are tonically inhibited by PIP_2_—like TRPV4 channels—and that G_q_PCR rapidly enhances the channel open probability. Both Piezo1 and TRPV4 are Ca^2+^-permeable cation channels; their activation could depolarize ECs and counteract Kir2.1-mediated hyperpolarization or promote NO release (5, 14, 15, 58). The implications of Piezo1 and TRPV4 sharing a common activation mechanism as well as similar functional consequences in brain ECs are not fully understood. One possibility is that simultaneous Piezo1 and TRPV4 activation synergistically counteract capillary hyperpolarization and bring CBF back to basal levels following hyperemia (5). Such synergism could follow different kinetics and temporal characteristics, extending signaling flexibility. Additionally, one channel’s activation could precede the other. Studies in cultured ECs proposed a mechanism by which Piezo1 activation leads to downstream TRPV4 activation which in turn sustains Ca^2+^ signals (59, 60). Whether a similar paradigm exists in brain ECs remains to be systemically tested. We, however, demonstrated previously that mechanically driven Ca^2+^ transients in retina CNS capillaries were insensitive to TRPV4 channel inhibition (6).

Our understanding of the mechanisms by which PIP_2_ alters Piezo1 function is evolving and requires further investigation. Direct binding of PIP_2_ to Piezo1 channel is one possibility. Recent molecular dynamics studies, supported with functional validation, have confirmed that major channel properties (e.g., resting configuration and pore structure) are determined by Piezo1/PIP_2_ interaction (31). Structural in silico studies have revealed several PIP_2_ binding sites within Piezo1 structure (35, 36). Many of these sites reside at disease-causing Piezo1 mutation locations, thus influencing channel inactivation kinetics and leading to an overactive channel. Structural modeling intriguingly suggested that the Piezo1 gain-of-function xerocytosis phenotype could be pathological through loss of PIP_2_ regulation, since the mutation interferes with PIP_2_ binding (35). Two lines of evidence support this suggestion. First, the ability of PIP_2_ to inhibit Piezo1 currents was weaker in Piezo1^EC-Mutant^ ECs (50% inhibition) than in ECs from nonmutant Piezo1 mice (~80 to 90%) (Fig. 4*E*). Second, structural study of human Piezo1 constructed a model in which the R2456 residue is critical in anchoring lipids at the channel hydrophobic pore. This binding allows fast channel inactivation, and the mutation severs such interaction (51). Further studies are needed to experimentally unravel PIP_2_ interaction with Piezo1 in brain ECs and reveal how Piezo1 mutations could interfere with such interaction.

PIP_2_ can alternatively influence Piezo1 activity by altering the mechanical properties of the plasma membrane. PIP_2_ regulates the adhesion of the plasma membrane to the actin cytoskeleton, as well as actin polymerization at the membrane influencing lipid domain organization (38, 61). Increased PIP_2_ levels enhance adhesion and membrane stiffness and potentially reduce membrane fluidity, effects that could influence Piezo1 function. Our data examining Piezo1 activation in ECs by negative pressure showed comparable responsiveness to mechanical stimulation, albeit at different open probability levels (*SI Appendix*, Fig. S1). Several lipids and proteins affect Piezo1 function (62–66) and could therefore contribute to our observations. For instance, cholesterol alters Piezo1 activity and could alter Piezo1 modulation by PIP_2_ (35, 67), suggesting Piezo1 dysregulation in pathological conditions with abnormal cholesterol levels. It should be noted, however, that Piezo1 activation by PIP_2_ depletion might not be a universal phenomenon. An earlier report suggested that PIP_2_ bioavailability is necessary for Piezo1 activity in a heterologous expression system (37). Several factors—such as cell type, experimental configuration, and the PIP_2_ form tested—could explain such discrepancies, highlighting potential differences in Piezo1 regulation in different systems.

Phosphoinositide levels are altered in disease. In the brains of AD patients, PIP_2_ is reduced (40, 41, 45), amyloid β increases PIP_2_ hydrolysis (68), and PLC-mediated PIP_2_ degradation is increased (69). PIP_2_ dysregulation also manifests in cSVD. Mouse models of cSVD showed that low PIP_2_ levels are driven by impaired PIP_2_ synthesis (27) or diminished PIP_2_ bioavailability due to phosphorylation modification by phosphatidylinositol-3-kinase (46). Experimental elevation of PIP_2_ in mouse models of AD or cSVD restored hyperemia, through the restoration of Kir2.1 channel function (27, 28, 46). In light of data presented here that Piezo1 function is dysregulated in 5xFAD and CADASIL mice and that such deficits can be corrected by PIP_2_, the rescue effects could be attributed to a double-hit effect with PIP_2_ simultaneously upregulating Kir2.1 activity and downregulating Piezo1 function. Consistent with enhanced Piezo1 function in AD mice, we recently reported that amyloid β (Aβ_1-40_) enhanced Piezo1 current activity via a mechanism that involved reactive oxygen species production (70) but we cannot exclude the possibility that Aβ peptides affect Piezo1 by lowering PIP_2_ levels (44). Future investigations will explore other conditions where PIP_2_ levels change and test whether Piezo1 activity changes. For example, *GNAQ* (gene encoding Gα_q_) mutations cause capillary malformation, and carrier patients show altered CBF that could be in part due to diminished PIP_2_ levels and increased Piezo1 activity (71–74).

In summary, our current results provide insights into the mechanisms underlying Piezo1 regulation in the brain endothelium. Specifically, we show that endothelial G_q_PCR signaling enhances Piezo1 channel function by depleting the inhibitor phosphoinositide PIP_2_. We further demonstrate that changes in endothelial PIP_2_ levels in different pathological conditions are associated with a Piezo1 channelopathy. Engineered mice carrying mutant endothelial Piezo1 channels suffered hyperemia deficits (5), and here we provide proof of concept that PIP_2_ injection corrects this impairment. Despite the weaker inhibition of mutant Piezo1 by PIP_2_, compared with wild-type channel, systemic treatment of mutant mice corrected blood flow defects. These data collectively suggest a potential therapeutic strategy in which the use of PIP_2_ could be a strategy to restore normal channel function and enhance brain blood flow. These findings establish the foundation for a therapeutic approach for improving CBF in conditions where Piezo1 activity is altered and could have impacts beyond brain blood flow control.

## Materials and Methods

### Animal Models.

All experimental protocols in this study are in accord with institutional guidelines and approved by the Institutional Animal Care and Use Committee (IACUC) of the University of Vermont (protocol X1-063). Adult (3- to 5-mo-old) male and female mice were group-housed on a 12-h light: dark cycle with environmental enrichment and free access to food and water. Mice used include wild-type C57BL/6J mice (Charles River Laboratories, USA). Tamoxifen-inducible, Piezo1^flox/flox^;*Cdh5*-Cre mice were engineered by crossing *Cdh5*-CreERT2 and floxed Piezo1^cx/cx^ mice in which exons 20 to 23 of the *Piezo1* gene are flanked by loxP sites (Strain: 029213, The Jackson Laboratory). Tamoxifen-inducible, Piezo1^cx/cx^; *Cdh5*-Cre mice were engineered by crossing *Cdh5*-CreERT2 and knock-in Piezo1^cx/cx^ mice (Model: 21617, Taconic Biosciences) in which floxed exons 45 to 51 are followed by a mutant Piezo1 copy (R2482H mouse mutation, equivalent to R2456H in human xerocytosis patients) (47, 75). For the induction of CreERT2, offspring homozygous with Piezo1^flox/flox^ or knock-in Piezo1^cx/cx^ and expressing *Cdh5*-Cre (i.e., Cre+) were treated with tamoxifen by providing tamoxifen citrate food (Envigo, TD.130859) for 7 consecutive days (daily tamoxifen intake, ~40 mg/kg). Tamoxifen treatment was followed by a washout period for 7 d before experimental intervention. The 5xFAD mouse strain used (6 months old, Strain: 34840, The Jackson Laboratory), B6SJL-Tg(APPSwFlLon,PSEN1*M146L*L286V)6799Vas/Mmjax), RRID:MMRRC_034840-JAX, was obtained from the Mutant Mouse Resource and Research Center (MMRRC) at The Jackson Laboratory, an NIH-funded strain repository, and was donated to the MMRRC by Robert Vassar, Ph.D., Northwestern University. Male and female 5xFAD mice and age-matched littermate controls were used for the study. The transgenic mouse lines *TgNotch3^WT^* (WT) and *TgNotch3^R169C^* (CADASIL) have been previously described (27). *TgNotch3^WT^* and *TgNotch3^R169C^* mice (FVB background) overexpress WT NOTCH3 and the CADASIL-causing NOTCH3^R169C^ mutant protein, respectively, to a similar degree and were used at an age of 6 months.

### Isolation of Brain Capillary ECs.

Mice were euthanized, in accord with the University of Vermont guidelines and approved by the IACUC (protocol X1-063), with 5% isoflurane inhalation followed by decapitation. Brains were isolated and placed in an ice-cold physiological salt solution (5 mM KCl, 140 mM NaCl, 2 mM MgCl_2_, 10 mM glucose, and 10 mM HEPES). Single capillary ECs were obtained from mouse brains by homogenizing somatosensory cortical tissue (∼10 to 15 mm^3^) in ice-cold isolation buffer (124 mM NaCl, 3 mM KCl, 2 mM CaCl_2_, 2 mM MgCl_2_, 1.25 mM NaH_2_PO_4_, 26 mM NaHCO_3_, and 4 mM glucose) using a Dounce homogenizer. Homogenates were passed through a 62-µm nylon mesh, and the filtered vasculature retained on the nylon mesh was eluted using a dissociation solution (55 mM NaCl, 80 mM Na-glutamate, 5.6 mM KCl, 2 mM MgCl_2_, 4 mM glucose, and 10 mM HEPES pH 7.3) containing protease (0.5 mg/mL), elastase (0.5 mg/mL; Worthington, MN), and 100 µM CaCl_2_. The suspension was incubated for 23 to 24 min at 37 °C, and then 0.5 mg/mL collagenase Type I (Worthington, MN) was added for additional 2 min incubation. The suspension was then filtered, and the enzymes were removed by washing the retained material with the isolation buffer. Single ECs were dispersed by triturating ~3 to 5 times using a glass Pasteur pipette. A few drops of the EC suspension were added to the recording chamber containing enzyme-free dissociation solution. Cell suspension was kept ice-cold, and cells were used within ∼5 h of dispersion.

### Patch-Clamp Electrophysiology.

Single-channel Piezo1 currents were recorded in the cell-attached configuration using an Axopatch 200B amplifier and a Digidata 1550B digitizer (Molecular Devices, USA) (6). Borosilicate glass pipettes (Sutter, USA), pulled with a Narishige puller and fire polished to a tip resistance of 4 to 5 MΩ with a Narishige microforge, contained a solution composed of 3 mM KCl, 10 mM HEPES, 137 mM NaCl, 1 mM MgCl_2_, 2 mM CaCl_2_, and 4 mM glucose (pH 7.35). The pipette solution was supplemented in some experiments with the Piezo1 channel activator Yoda1 (5 μM). To minimize the contribution of other EC ion channels to the recorded current, GSK2193874 (0.1 μM, TRPV4 blocker) and BaCl_2_ (100 μM, Kir2.1 blocker) were included in the pipette solution. The bath solution consisted of 140 mM KCl, 10 mM HEPES, 1 mM MgCl_2_, 2 mM CaCl_2_, and 4 mM glucose (pH 7.35). Recordings were made at −50 mV after a giga-ohm seal was achieved. The presence of the Piezo1 channel in the patch was confirmed by observing ∼1 pA openings that were mechanically triggered by negative pressure (−10 mmHg). The dwell times (open and closed) were calculated using Clampfit 11.2 analysis software by fitting binned dwell-time histograms. The average dwell-time values were calculated from all events in the category or level of the current event (i.e., level 0, closed; level 1, open). A subset of experiments were performed in the outside-out configuration (voltage-clamp mode, patches held at −70 mV) as done before (7, 31). The bath solution was composed of 135 mM NaCl, 10 mM HEPES, 4 mM KCl, 2 mM CaCl_2_, 1 mM MgCl_2_, and 10 mM glucose (pH 7.4 using NaOH). The pipette solution consisted of 145 mM KCl, 1 mM MgCl_2_, 0.5 mM EGTA, and 10 mM HEPES (pH 7.2 using KOH). All experiments employing PIP_2_ used the water-soluble sodium salt of diC16-PIP_2_ (Item: 10008115, Cayman Chemical; Molecular Formula C_41_H_78_O_19_P_3_.3Na). Mechanical activation of Piezo1 was achieved in the cell-attached configuration by applying negative pressure steps (0, −5, −10, -15, -20, and −25, mmHg), calibrated using a pneumatic transducer (Fluke), onto the patch. The minimum recording duration for data inclusion/analysis was 300 s.

### LSCI.

Mice were first anesthetized by isoflurane inhalation (4 to 5% for induction, 1.5 to 2% for maintenance), followed by intraperitoneal injection of α-chloralose (50 mg/kg) and urethane (750 mg/kg). To prevent excess lung secretions and breathing difficulty during experiments, mice were given atropine (0.05 mg/kg, subcutaneous). For the duration of the procedure and imaging, we used a thermometer and feedback-controlled heating blanket (RWD) to maintain body temperature at 37 °C. Mice were shaved and positioned in a stereotaxic frame to immobilize the head. The surface tissue overlaying the cranium was removed surgically, the skull was exposed, thinned using a microdrill, and covered with agarose and a glass coverslip (thickness size 0) (76, 77). A custom-built LSCI setup was used for imaging blood flow dynamics in real-time through the thinned skull, as we did before. The setup included delivering near‐infrared laser (785 nm, LP785‐SAV50, Thorlabs) controlled using a diode driver (CLD1010LP, Thorlabs). Backscattered light was recorded using a zoom imaging lens (VZM 450i, Edmund Optics) mounted on a near infrared-sensitive CMOS camera (Basler ace acA2000-165 μm NIR, Basler, Germany). LSCI data were analyzed by calculating an arbitrary blood flow index which represents 1/K^2^ where K is contrast. Baseline and stimulation recordings were made over a field of view 8 × 8 mm with 1024 × 1024 pixels with a frame rate of 50 Hz (50 frames per second [fps]) or 15 fps for hypercapnia experiments. Data were acquired using custom-built acquisition software and processed using spatial speckle contrast analysis to get a high temporal resolution. Customized MATLAB scripts were used to process and analyze LSCI data. Differential hyperemia mapping was routinely used to locate the region with maximal response during whisker stimulation (i.e., barrel cortex). Whiskers were stimulated with air puffs (using a custom-built air stimulator) for 5 s at 5 Hz. Hypercapnia was induced by mixing the inspired oxygen-enriched air with 10% CO_2_ using a flowmeter (MF5700, ATO). diC16-PIP_2_ (0.5 mg/Kg body weight) or saline were injected into the retroorbital cavity, and recordings were made before and 20 min after injection.

### Reagents.

PGE_2_ was purchased from Sigma-Aldrich (USA), and all other reagents were purchased from Cayman Chemicals and Fisher.

### Statistical Analysis.

Data are expressed as mean ± SEM, unless mentioned otherwise. Experiments were performed in a random manner (animals, pharmacological treatments). Statistical tests (indicated in figure legends) were performed using GraphPad 10 software. *P*-values < 0.05 were considered significant.

## Supplementary Material

Appendix 01 (PDF)

## Data Availability

All study data are included in the article and/or *SI Appendix*.
