# Supplementary material for: PIP2 corrects an endothelial Piezo1 channelopathy
Source: Proc Natl Acad Sci U S A. 2025 Dec 23;122(52):e2522750122. doi: 10.1073/pnas.2522750122 (PMC12772150; doi:10.1073/pnas.2522750122)
Supplement: Supplementary file 1 — Appendix 01 (PDF) [file pnas.2522750122.sapp.pdf]

SUPPLEMENTARY INFORMATION (SI)

PIP_2 Corrects an Endothelial Piezo1 Channelopathy

Ahmed M. Hashad, Mohammad M. Abd-Alhaseeb, Xin Rui Lim, Natalia M. Mathieu, Osama F. Harraz*

Department of Pharmacology, Larner College of Medicine, Vermont Center for Cardiovascular and Brain Health, University of Vermont, Burlington, Vermont

***Correspondence**: Osama.Harraz@uvm.edu

Classification:

Major: Biological Sciences.

Minor: Neuroscience

Key words: Brain, Endothelium, G_qPCR, Piezo1, PIP_2, Alzheimer's, CADASIL, Piezo1 GOF, Patch clamp electrophysiology, Functional hyperemia

Supplementary Figure. 1

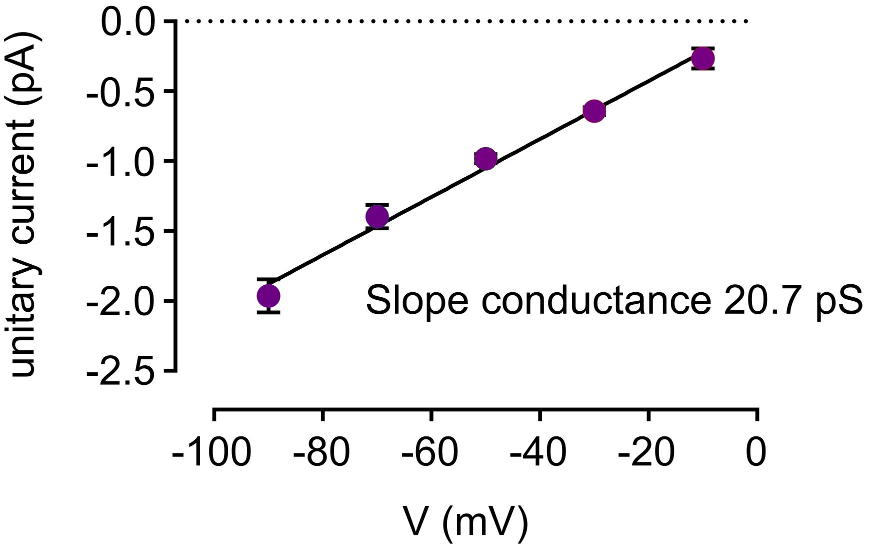

Suppl. Fig. 1. Unitary conductance of evoked currents in capillary ECs.

Unitary current-voltage relationship plotted using averaged unitary currents measured at the following voltages (mV): −10, −30, −50, −70, and −90 in the cell-attached configuration and in the presence of wortmannin (50 μM). Conductance obtained from the slope was 20.7 pS. Data were obtained from 4 ECs isolated from 2 C57 mice.

Supplementary Figure. 2

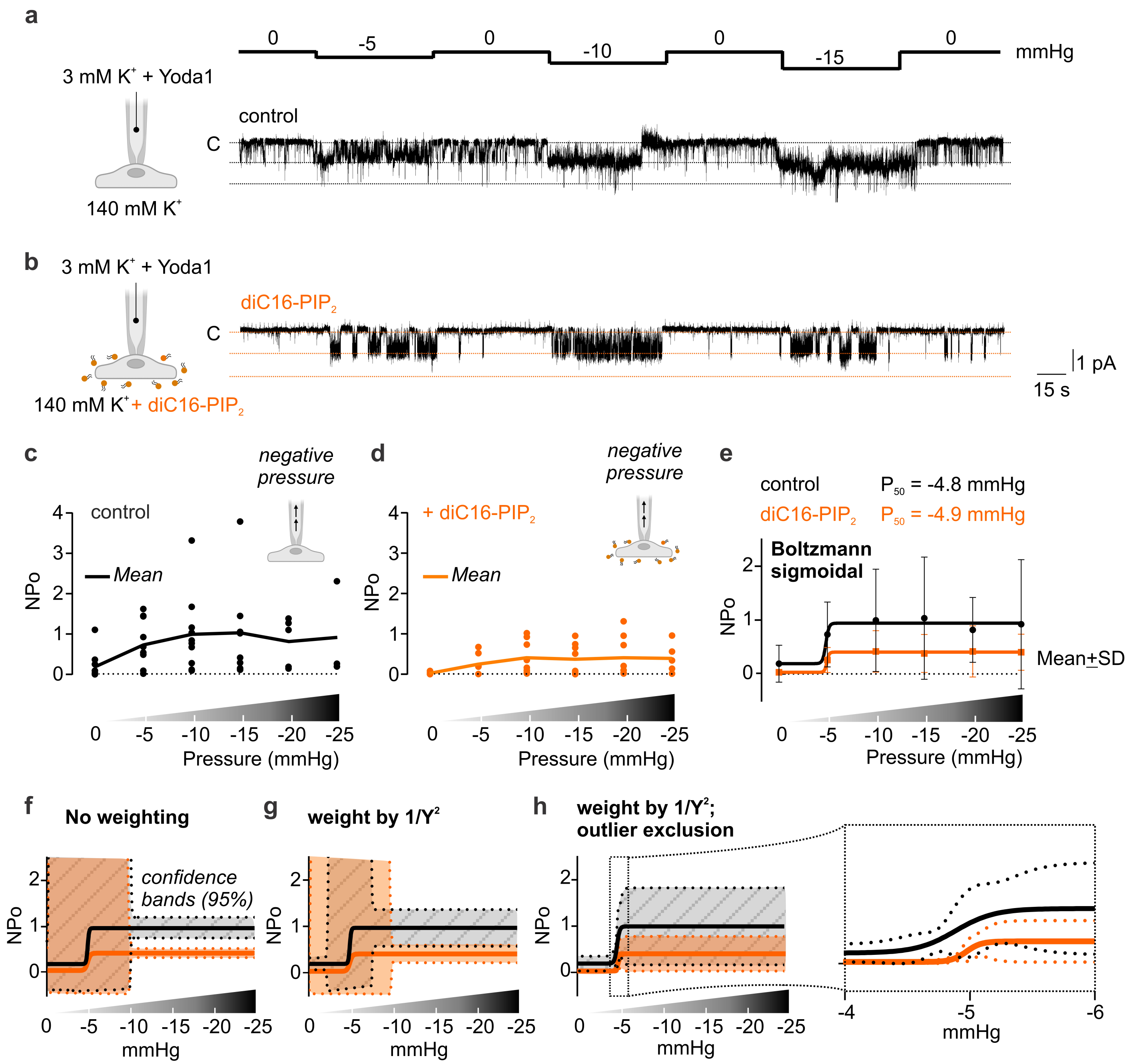

P_{50} (mmHg)	-4.847	-4.948
R^2	0.9429	0.9920
Adj. R^2	0.8572	0.9801

P_{50} (mmHg)	-4.878	-4.973
R^2 (weighted)	0.2474	0.4440
Adj. R^2 (weighted)	0.1949	0.4023

P_{50} (mmHg)	-4.886	-4.972
R^2 (weighted)	0.2015	0.3822
Adj. R^2 (weighted)	0.1094	0.2664

Suppl. Fig. 2. Mechanical activation of EC Piezo1 channel.

Mechanosensitivity of Piezo1 channel in C57BL/6J ECs. Traces show Piezo1 channel openings induced by negative pressure steps (-5, -10, -15 mmHg, with 0 mmHg intervals in between) in the absence (**a**) or the presence (**b**) of diC16-PIP_2 (10 µM). (**c, d**) Scatter plots and mean values (*lines*) of Piezo1 NP_O at different pressure steps while diC16-PIP_2 present or absent. (**e**) Averaged Piezo1 activity (depicted as mean ± standard deviation [SD]) and Boltzmann sigmoidal fits in response to rising negative pressure onto the patch (0 to -25 mmHg, 5 mmHg increments), while diC16-PIP_2 was absent or present. P_{50} denotes pressure

eliciting half-maximal activation. Currents were recorded at -50 mV in the presence of 5 μM Yoda1 (0 mmHg: n=10/6 control, n=8/6 PIP_2; -5 mmHg: n=10/6 control, n=7/6 PIP_2; -10 mmHg: n=10/6 control, n=8/6 PIP_2; -15 mmHg: n= 9/6 control, n=8/6 PIP_2; -20 mmHg: n=6/6 control, n=8/6 PIP_2). (**f**) Sigmoidal fits (nonlinear regression, Boltzmann function) of Piezo1 activity in response to pressure along with the corresponding confidence bands (no weighting, confidence level 95%). (**g, h**) Weighted ($1/Y^2$) nonlinear regression accounting for the number of replicates and the SD, without (***g***) or with (***h***) outlier exclusion (Q=1). Respective estimated P_{50}, R^2, and adjusted R^2 values are shown in the bottom tables. The right inset in *h* shows the fits and confidence intervals at pressure values between -4 and -6 mmHg.

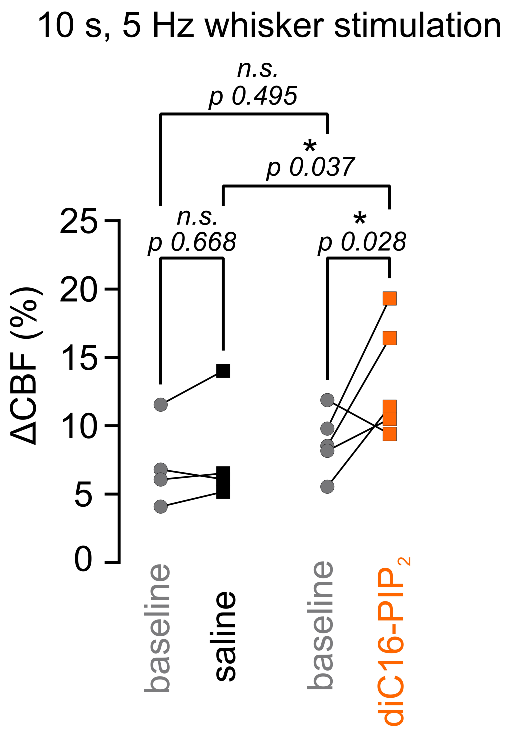

Supplementary Figure. 3

Suppl. Fig. 3. PIP$_2$ enhances 10s whisker stimulation induced FH in Piezo1$^{EC\text{-}Mutant}$ mice.

Maximal FH responses in Piezo1$^{EC\text{-}Mutant}$ mice before and after saline (n=4 mice) or PIP$_2$ injection (n=5 mice). Whiskers were stimulated with air-puffs (10 s at 5Hz). Two-way ANOVA with Fisher's LSD test for multiple comparisons (*P < 0.05; *n.s.* denotes not significant).
